# Automatic Classification of Users’ Health Information Need Context: Logistic Regression Analysis of Mouse-Click and Eye-Tracker Data

**DOI:** 10.2196/jmir.8354

**Published:** 2017-12-21

**Authors:** Wenjing Pian, Christopher SG Khoo, Jianxing Chi

**Affiliations:** ^1^ Decision Sciences Institute School of Economics & Management Fuzhou University Fuzhou China; ^2^ Wee Kim Wee School of Communication & Information Nanyang Technological University Singapore Singapore; ^3^ College of Communication Fujian Normal University Fuzhou China

**Keywords:** information-seeking behavior, social media, Internet, consumer health information, medical informatics

## Abstract

**Background:**

Users searching for health information on the Internet may be searching for their own health issue, searching for someone else’s health issue, or browsing with no particular health issue in mind. Previous research has found that these three categories of users focus on different types of health information. However, most health information websites provide static content for all users. If the three types of user health information need contexts can be identified by the Web application, the search results or information offered to the user can be customized to increase its relevance or usefulness to the user.

**Objective:**

The aim of this study was to investigate the possibility of identifying the three user health information contexts (searching for self, searching for others, or browsing with no particular health issue in mind) using just hyperlink clicking behavior; using eye-tracking information; and using a combination of eye-tracking, demographic, and urgency information. Predictive models are developed using multinomial logistic regression.

**Methods:**

A total of 74 participants (39 females and 35 males) who were mainly staff and students of a university were asked to browse a health discussion forum, Healthboards.com. An eye tracker recorded their examining (eye fixation) and skimming (quick eye movement) behaviors on 2 types of screens: summary result screen displaying a list of post headers, and detailed post screen. The following three types of predictive models were developed using logistic regression analysis: model 1 used only the time spent in scanning the summary result screen and reading the detailed post screen, which can be determined from the user’s mouse clicks; model 2 used the examining and skimming durations on each screen, recorded by an eye tracker; and model 3 added user demographic and urgency information to model 2.

**Results:**

An analysis of variance (ANOVA) analysis found that users’ browsing durations were significantly different for the three health information contexts (*P*<.001). The logistic regression model 3 was able to predict the user’s type of health information context with a 10-fold cross validation mean accuracy of 84% (62/74), followed by model 2 at 73% (54/74) and model 1 at 71% (52/78). In addition, correlation analysis found that particular browsing durations were highly correlated with users’ age, education level, and the urgency of their information need.

**Conclusions:**

A user’s type of health information need context (ie, searching for self, for others, or with no health issue in mind) can be identified with reasonable accuracy using just user mouse clicks that can easily be detected by Web applications. Higher accuracy can be obtained using Google glass or future computing devices with eye tracking function.

## Introduction

### Background

Searching for health information on the Internet is common. A national survey in Scotland found that in 2015, 68.4% (379/554) survey respondents had previously searched for health information on the Internet [1]. Another telephone survey conducted in 2012 in the United States found that 59% (1778/3014) participants used the Internet to find various types of health information, such as symptoms, treatment, dietetic information, and drug information [2]. Web-based health information was mainly used for self-diagnosis, communication with doctors, and for keeping fit. A study in Hong Kong found that users had accessed various types of health information on social media sites (eg, Facebook, Twitter, and discussion forums) to obtain health information [3].

There are various types of health information available on the Internet from general medical terms to users’ experience of chronic diseases and drugs [4]. It takes time and effort for Internet users to find health information that is relevant to their situation while filtering out non-relevant information. It has been found that as the time spent in searching for health information increases, user anxiety about the health issue may also increase [5,6]. Health information websites and applications should therefore be designed to provide users with more relevant health information while reducing the users’ time spent on filtering out nonrelevant information. Unfortunately, most health information websites provide static health information content to all users, with no attempt to customize or personalize the information to individual needs. Users have to exert substantial effort in skimming and filtering out nonrelevant health information.

Previous studies have found that users seeking health information on the Internet can be categorized into the following three types of user health information need contexts: searching for the users’ own health issue, searching for someone else’s health issue (ie, for a family member or friend), and browsing with no particular health issue in mind [4,7]. When browsing for own health issue, users tend to make use of case-based relevance judgment to match the information with their own health condition (eg, personal history of disease and personal symptoms). When browsing for other people’s health issue, they tend to focus on general information (eg, medical terms, drug names, and drug description). When browsing with no particular health issue in mind, users tend to focus on issues of general interest, current hot topics, and unusual diseases. Users in these three types of health information contexts make use of different criteria in assessing the relevance and usefulness of health information that they encounter on the Internet [4]. Understanding the relations between users’ searching or browsing patterns on health information websites and the types of user information need can help website and Web application developers to customize health information for particular categories of users and to increase the likelihood of relevance of the information [8].

This study investigated particular types of user browsing patterns, in particular different types of eye movement durations, and the associations between them and the three types of user health information context. The eye movements were recorded by a Tobii T60 series eye tracker (Tobii AB, Sweden). As most Internet users access health information websites anonymously, personal profile information is unavailable to the website. Hence, mouse click patterns and, in the future, eye movement data are possible ways of identifying the category of user to achieve some customization of health information. Compared with user browsing patterns such as reading time and mouse clicks [9-14], eye movement data provide more insights on user’s relevance decision on whether health information is relevant [4,7].

This study was conducted on a particular health discussion forum, HealthBoards.com. It was chosen as the research platform because it contains various types of health information [4] and has a large number of users and good coverage of health topics.

A typical health discussion forum has a 3-stage user interface interaction structure. Table 1 lists the three types of screens displayed by the system in column 1 and the corresponding user action in column 2. Stage 1 displays the search screen in which the user can either enter a search query or browse a hierarchical menu of health issues to select a category. Stage 2 displays a summary result page of post surrogates (mainly post titles) retrieved for the search query or for the health category selected. Clicking on a post surrogate will display a detailed post screen (Stage 3) with the post content together with comments from other users.

In stage 2 summary result screen, the expected user action is to scan the post surrogates displayed to choose a post to read in detail by clicking on it. In stage 3 detailed post screen, the expected user action is to read the detailed post content. User browsing of a health discussion forum can thus be divided into following the three stages: (1) specifying a search query or browsing the hierarchical menu of health problem categories to select a category, (2) scanning the summary result screen of post surrogates, and (3) reading the detailed post content. Within stage 2 and 3, users can either (a) examine the text closely (indicated by eye fixations) or (b) skim the text to locate the next piece of text to examine (indicated by eye saccades and quick eye movements) [15,16]. This study adopted this framework of user interaction with an information system from previous studies [4,7] to analyze different types of browsing behavior on a health discussion forum.

Bivariate analysis was carried out to identify associations between different eye movement durations and the types of user health information context. Then, multinominal logistic regression analysis was used to develop classifiers to predict the types of health information context from the eye movement durations. The following three types of predictive models were developed: model 1 used only the time spent in scanning the summary result screen and reading the detailed post screen, which can be determined from the user’s mouse clicks; model 2 used the examining and skimming durations on each screen, recorded by an eye tracker; and model 3 added user demographic and urgency information to model 2.

**Table 1:** Stages of user browsing and searching in a health discussion forum.

Type of screen displayed by the system	Expected user action
Stage 1. Search screen and hierarchical menu of health problem categories	(1) Enter search query or browse the hierarchy of health problem categories to select a category
Stage 2. Summary result screen displaying a list of post surrogates (mainly post titles) retrieved	(2) Scan the list of post surrogates to select one to click on. This stage can be divided into 2 types of subactions: (a) Examine individual post surrogates closely (indicated by eye fixations) and (b) Skim the list of post surrogates quickly (indicated by quick eye movement and eye saccades)
Stage 3. Detailed post screen displaying detailed post content and user responses to the content	(3) Read the detailed post content and user responses to the content. This stage can again be divided into two types of subactions: (a) Examine and comprehend the content (eye fixations) and (b) Skim the content (quick eye movement)

### Prior Work

This section reviews the following three areas of related research: studies on different types of health information needs during health information seeking, methods of tailoring and personalizing health information by websites and applications, and studies on eye movement patterns in relation to user characteristics.

#### Types of Health Information Needs

Information need has been broadly characterized as the perceived need for information that leads to someone using an information retrieval system [17]. It is the motivation for the user to search or browse for information to address a particular issue or purpose [18]. Hence, users’ information needs are related to what particular information they want to find. In health information seeking, users want to find different types of health information to address different health information needs. For example, users with coronary syndromes were found to seek information for symptom management as well as for long-term survival [19], whereas users with cancer want to find detailed information of the illness and potential treatments [20]. With the proliferation of social media sites, users are increasingly posting health-related questions on these sites as well as reading other users’ posts to address different information needs. Users with diabetes were found to search social media sites for information to manage their condition [21].

In the abovementioned and many other studies of health information needs, the focus was to identify the content of these needs, whereas the context of the information need has not gained much attention [22]. A few studies have acknowledged that users do seek information on behalf of family and friends [2,23]. As early as 2000, a survey in the United States found that about 50% of Web-based health information seekers searched for health information on behalf of someone else [24]. Users have been found to search for health information for family members and loved ones including children [25,26]. In addition, users have been found to find useful health information serendipitously [4,27]. However, there has been no detailed study of health information–seeking behavior on behalf of other people or casual browsing of health information sites. The exception is the recent study by Pian et al [4] who found that users who sought information relevant to other people’s health issue focused on different relevance criteria than users seeking information for own health issue. Users seeking health information for their own self focused on detailed symptoms and patient experience, whereas users seeking information for others focused on basic medical knowledge and basic concepts. On the other hand, users browsing with no particular health issue in mind focused on hot topics and unusual cases. This suggests that websites and Web applications should distinguish between these three types of health information need context and attempt to provide tailored and personalized health information for these categories of users.

#### Tailored and Personalized Health Information

Tailored health information has been characterized as specifically designed health information content for specific people based on their unique needs and interests [28]. Tailored health information is related to tailored health communication, aimed at applying specific information and behavior strategies to a particular person to facilitate behavioral change, such as smoking cession [28]. Tailored health information has been shown to be effective in increasing user’s knowledge and understanding of health issues and influencing health behavior change [29]. It is also effective in making health information more relevant to the audience [30]. For instance, tailored Web-based health-related message on breast cancer’s association with smoking was shown to increase the awareness of boys and girls on the risk and to stimulate their seeking specific health information rather than general health information [31].Web-based tailored information on alcohol was shown to be effective in changing unhealthy drinking patterns for adults in the Netherlands [32]. Hence, tailored health information has more impact on users’ understanding and knowledge because the information is more relevant to the users’ situation and needs [33].

Personalization of websites has been characterized as a process to collect user information during interaction for delivering appropriate services and content. The purposes of personalization include the following: serve user better by predicting user’s needs, make the interaction efficient, and provide good experience to encourage repeat visits [34]. The personalization of health information websites can provide more benefits for health information seekers, such as automation and accessibility, extended medical knowledge, user-friendly health-related language (especially for the layman), and patient privacy control [35].

Tailoring of health communication and personalization of health information are related. The former incorporates information strategies and behavioral strategies and often has the ultimate goal of changing health-related behavior by intervention. The latter focuses on providing user-specific information and makes the interaction between user and health information service more effective. Hence, Rimer and Kreuter [28] stated that “Tailored health communications usually are personalized, but merely being personalized is not sufficient to consider them tailored health communications.” The results of this study carry implications for both tailoring of health communication and personalization of health information, as an earlier study has found that users seeking health information for self, for others, or with no specific health issue are interested in different kinds of health information. Thus, detecting the user’s health information need context by the website as part of personalization makes it possible for the system to tailor specific types of information that is more likely to be of interest to the user. However, this study does not address the issue of influencing the health information seeker’s behaviors.

#### Eye Movement Patterns in Relation to User Characteristics

Eye movements are thought to be related to people’s cognitive process or cognitive perspective [4,36]. Although eye movements cannot reveal the process or perspective directly, they can serve as a reference for understanding and inferring the related cognitive process [37]. A few studies have been conducted in this area.

Buscher et al conducted studies to investigate the relation between different types of eye movements and user’s relevance feedback [16,38]. In this study, the researchers defined reading and skimming as different types of eye movements and use them as indicators of user’s relevance feedback. They assigned each type of eye movement a different score and calculated the cumulative scores of both the total examining and skimming found in user’s browsing of a particular document. Then, the ratio of reading score to skimming score for a particular document was found to be positively correlated with users’ relevance feedback. When the ratio increased, the document was more likely to be thought as relevant.

Pian et al analyzed the health information content focused on by users with the three types of health information need context [4,7]. Content analysis was carried out of what users focused on when browsing a health discussion forum, using users’ eye movement data. They divided the browsing process into following two stages: (1) scanning the summary results screen of post surrogates and reading the detailed post content screen (2) and analyzing the types of information that users’ eyes fixated on and coding them into different categories of health information. They found that users seeking information for their own health issue focused on case-based health information, including personal disease history, symptoms, and personal feelings. Users seeking information on a health issue of a family member or friend focused on general health information, including basic medical knowledge, terms, and treatments. In contrast, users browsing with no particular health issue in mind focused on issues of general interest, current hot topics, and rare diseases.

Another study investigated the relation between eye movements and users’ background knowledge acquisition process [39]. The study developed three types of eye movement measures: Lexical Access Duration Excess (a duration above 113 ms indicates acquisition of word meaning), perceptual span (the distance that reflects the spacing of fixations and describes the length of text that users take as a unit), and reading speed. They found that these three measures were correlated positively with users’ domain knowledge level.

There were still other studies focusing on the relation between users’ eye movement pattern and users’ cognitive perspectives. A study was conducted to investigate the associations between users’ eye movements and their cooking interest [40]. Another study investigated the connection between user’s health literacy and their preference of medical illustration [41]. Other studies have investigated user’s viewing of Web-based commercial products and potential employees [42,43]. However, these studies did not attempt to develop predictive models. In this study, we sought not only to find the associations between users’ eye movement patterns and their types of health information need context but also to develop a logistic regression model to identify whether a user is seeking health information for self, for others, or with no particular health issue in mind.

## Methods

### Study Design and Data Analysis

This study was divided into two parts. The first part sought to find out the associations between the three types of user health information contexts and browsing durations (eye movement durations), and the second part developed predictive models to identify the user’s health information need context from eye movement and other measures.

**Figure 1:**
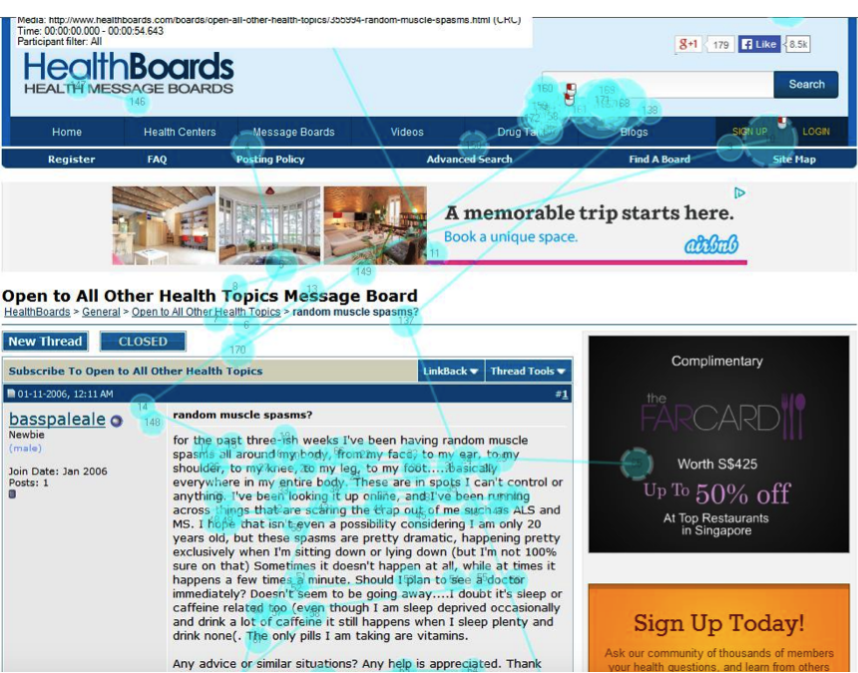
Screenshot of examining and skimming in the detailed post page.

We have earlier described the 3-stage framework of user interaction with the discussion forum as summarized in Table 1. The focus of the analysis is on stage 2 when the user scans the list of post surrogates in the summary result screen and stage 3 when the user reads the detailed post content. In both the scanning and reading stages, users exhibit the following two types of eye movements: examining (eye fixation, indicating close reading) and skimming (quick eye movements). Figure 1 shows a screenshot of a detailed post content screen with these two kinds of eye movements. The round spots represent examining behavior (eye fixation), and the size of the spot represents the duration of the eye fixation. The lines between two examining spots represent skimming behavior, and its duration is measured by the difference between the timestamp of the user leaving the first examining spot and entering the second examining spot. All the information was stored in the eye-tracking system and was exported to a Microsoft Excel spreadsheet for further analysis.

The user’s cumulative examining duration on a particular webpage of the health discussion forum was calculated by adding all the durations of individual examining spots within the page. The cumulative skimming duration was calculated by adding all the skimming durations. Then, a particular user’s average examining duration and skimming duration were calculated by averaging all the cumulative examining and skimming durations across the webpages viewed by the user. The average examining duration and average skimming duration for a user were calculated separately for the stage of scanning the summary result screen and the stage of reading the detailed post content.

To develop the predictive model to identify the particular type of user health information context, we developed 3 models using multinominal logistic regression analysis. Model 1 used the duration of scanning the summary result screen of post surrogates and the duration of reading the detailed post content screen to predict the user’s type of health information context. These durations can be recorded from the user’s mouse clicks of entering or leaving a particular webpage within the health discussion forum. Hence, it does not require detailed eye movement data recorded by the eye tracker but only the hyperlink click times that are available to the Web application system. Model 2 used the examining and skimming durations within the two browsing stages in a stepwise multinominal logistic regression to develop a model to predict the user’s health information context. Model 3 used the detailed eye movement durations, the user’s demographic information, and the urgency level of the health information need to predict the user’s health information context. The participants in the study were asked to indicate the urgency of the health information need on a 1 to 7 Likert-like scale, at the beginning of the experiment session. The variables used in the 3 models are listed in Textbox 1.

**Variable lists for 3 predictive models.**

Model 1

- Duration of scanning stage
- Duration of reading stage

Model 2

- Duration of scanning stage
- Duration of reading stage
- Examining duration at scanning stage
- Skimming duration at scanning stage
- Examining duration at reading stage
- Skimming duration at reading stage
- The ratio of examining to skimming at scanning stage
- The ratio of examining to skimming at reading stage

Model 3

- Duration of scanning stage
- Duration of reading stage
- Examining duration within scanning stage
- Skimming duration within scanning stage
- Examining duration within reading stage
- Skimming duration within reading stage
- The ratio of examining to skimming at scanning stage
- The ratio of examining to skimming at reading stage
- Gender
- Age
- Education
- Level of urgency

This study adapted the following steps used in previous eye-tracking studies [4,7]:

1. Brief the participant on the aim of the study and ask the participant to sign the informed consent form.
2. Ask the participant to describe a particular health information need. If the participant is searching for own health issue or other’s health issue, ask for details of the topic. If the participant does not have a particular topic to browse, ask the participant to browse the health discussion forum for fun. Ask the participant for the following demographic information: age, gender, education level, and urgency level of health information need (1 to 7 Likert-like scale).
3. Introduce the eye tracker machine to the participant and complete the eye tracker machine calibration test. This calibration is used to adjust the machine to a particular participant. If the calibration was not successful, the participant was excluded from the study.
4. Ask the participants to browse for relevant information or for fun on the predefined health discussion forum. No time limit is set for the participant to browse. The participant can browse until the participant is satisfied or wants to stop.
5. Replay the video of the participant’s browsing, and ask the participant to comment on why he or she focused on particular texts and why the participant took a longer or shorter time on different pages.

### Study Setting and Sample Size

This study was conducted on a particular health discussion forum, HealthBoards.com. It was selected because it had the highest number of registered users and the highest number of posts. The list of candidate health discussion forums and the basic statistics for HealthBoards.com are given in Textbox 2 and Textbox 3, respectively.

A total of 80 participants signed the informed consent, and 74 participants passed the eye tracker machine calibration test. The demographic profile of the participants is given in Table 2.

### Study Population

The study population can be characterized as laypersons in Singapore who were not health professionals and who did not have a critical or severe disease. Health professionals and critically ill people were not explicitly excluded from the study. It is just that the participants who volunteered did not include such people. As people with critical or severe diseases and health professionals are expected to exhibit different health information–seeking behaviors [4], the results of this study should not be generalized to them. Separate studies focusing on these two categories of people are needed. For determining critical and severe diseases, we consulted the list of critical illnesses listed on the website of the Life Insurance Association of Singapore, including cancer, AIDS, and coma [44].

### Sampling Technique and Ethics

Convenience sampling was used in this study to recruit research participants. Invitation emails were sent out to students and staff of Nanyang Technological University, and invitation phone calls were made to the researchers’ friends outside of campus. Besides, posters were posted on various notice boards on campus to recruit participants. Participants were given SG $15 for participating.

The institutional review board of Nanyang Technological University approved the study before data collection. All the research participants were required to read and sign the informed consent before they took part in this study.

**List of candidate health discussion forums.**

Discussion forums on the patient website

- Health Forum
- HealthBoards.com
- eHealth forum
- netdoctor
- Consumers of Health Forum of Australia
- Mental Health Forum
- PatientsLikeMe
- Health Informatics Forum

**Basic statistics for HealthBoards.com.**

- Number of registered users: over 1,100,000 registered users as of January 2017; the forum with the second largest number of registered users was PatientsLikeMe with 500,000 registered users
- Number of posts: 879,065 threads and 4,874,692 posts and replies
- Number of subsections on particular health conditions and problems: over 280 subsections
- Number of daily online users: 3000+
- Ranking: Number 1 health forum in Yahoo Health search

**Table 2:** Demographics of research participants (N=74).

Demographics		n (%)
**Nationality**		
	Chinese	32 (43)
	Singaporean	34 (46)
	Others	8 (11)
**Education level**		
	Undergraduate	22 (30)
	Master’s degree	32 (43)
	PhD	20 (27)
**Occupation**		
	Full-time student	34 (46)
	Part-time student	14 (19)
	University staff	16 (22)
	Working adults	10 (13)
**Age in years**		
	18-20	10 (14)
	>20-30	30 (40)
	>30-40	25 (34)
	>40-50	9 (12)
**Gender**		
	Male	35 (47)
	Female	39 (53)
**Type of heath information context**		
	Browsing for self	25 (34)
	Browsing for others	23 (31)
	Browsing with no issue in mind	26 (35)

## Results

### Associations Among Browsing Durations and Demographic Factors

Associations between different browsing durations and different human factors were analyzed by correlation analysis, by analysis of variance (ANOVA),and post hoc analysis. ANOVA analysis was used to analyze differences among more than two categories. It was followed by post hoc analysis to analyze differences between each pair of categories, as three categories of health information context were investigated.

Bivariate correlation analysis was carried out among the following independent variables: the different browsing durations, demographic information, and urgency level. Recall that browsing of the health discussion forum in this study is divided into two stages: *scanning* the summary results screen (displaying post surrogates) and *reading* the detailed post content screen (displaying a post content and comments from users). Each stage is subdivided into *skimming* (quick eye movements) and *examining* (eye fixation). The scanning duration was highly correlated with scanning-skimming duration (Pearson *r*=.92), indicating that scanning post surrogates is associated with skimming (rather than examining). On the other hand, the reading duration was highly correlated with reading-examining duration (*r*=.89), indicating that reading post content is associated with examining rather than skimming.

Looking at demographic variables, age was found to be positively correlated with reading duration, reading-examining duration, scanning-examining/skimming ratio, and reading-examining/skimming ratio (all significant at *P*<.01) (ie, mainly examining durations). Age was negatively correlated with scanning-skimming duration and reading-skimming duration (*P*<.011) (ie, mainly skimming durations). In summary, older people do more examining, whereas younger people do more skimming.

The urgency level of the information need was positively correlated with reading duration, reading-examining duration, and scanning-examining/skimming ratio (*P*<.016) (ie, mainly examining durations). It was negatively correlated with scanning-skimming (*P*<.01) and reading-skimming (*P*<.05) durations (ie, mainly skimming). Clearly, people with greater health urgency do more examining, whereas people with lower health urgency do more skimming.

ANOVAs were carried out to find out whether nationality (Singaporean, Chinese, or others), education level (undergraduate, postgraduate, and PhD), and gender were significant factors in explaining differences in the browsing durations.

Nationality was found significant in explaining differences in scanning-examining/skimming ratio (*F*_73_=7.5, *P*<.001). The ratio was lowest for Chinese nationals, medium for other nationalities, and highest for Singaporean. This means that Chinese nationals do more skimming than examining when scanning post surrogates, compared with other nationalities.

Education level was a significant factor for explaining differences in reading duration (*F*_73_=4.2, *P*<.05), reading-examining duration (*F*_73_=5.1, *P*<.01), and reading-examining/skimming ratio (*F*_73_=6.4, *P*<.01). The three dependent variables have higher values as the education level increases, that is, higher education is associated with longer examining time during the reading of post content.

Gender was not found to be significant.

### Association Between User Health Information Need Context and Browsing Durations

ANOVAs were performed to find out whether the between-group differences (for the three types of health information context) were significant for the different browsing durations. Table 3 shows that all the between-group differences were significant (*P*<.001) for all the browsing durations. However, the durations that obtained the highest *F* scores were as follows: scanning, scanning-skimming, and scanning-examining.

As the focus of this study was the associations between types of user health information context and different browsing durations, post hoc tests were conducted to identify significant differences between every two groups of participants with different health information contexts. Group 1 refers to the category of seeking health information for self, group 2 refers to the category of seeking for others, and group 3 refers to the category of browsing with no health issue in mind. Post hoc tests determine whether there were significant differences between groups 1 and 2, groups 1 and 3, and groups 2 and 3.

For scanning post surrogate stage, it was found that participants browsing with no particular issue (group 3) had much longer skimming duration than the other two groups of participants (*P*_1,3_<.001and *P*_2,3_<.001). However, there was no significant difference for skimming duration between participants browsing for self (group 1) and others (group 2) (*P*_1,2_=.744).

For the examining duration, post hoc tests showed that participants browsing with no particular issue had shorter examining duration than participants browsing for self and with no issue (*P*_1,3_<.001 and *P*_2,3_<.001) and there was no significant difference for this examining duration between participants browsing for self and with others (*P*_1,2_=.074). The details are given in Table 4.

**Table 3:** Results of analysis of variance (ANOVA) test of between-group differences.

Eye movement measures		ANOVA test	
		*F* (degree of freedom=73)	*P* value
**Scanning post surrogate duration**		34.82	<.001
	Skimming duration at post surrogate stage	32.06	<.001
	Examining duration at post surrogate stage	40.01	<.001
**Reading detailed post content duration**		24.67	<.001
	Skimming duration at detailed post stage	12.18	<.001
	Examining duration at detailed post stage	27.19	<.001

**Table 4:** Post hoc test results for different durations.

Eye movement measures		Post hoc test					
		Group 1: Browsing for self (N=25), mean	Group 2: Browsing for others (N=23), mean	Group 3: Browsing with no particular issue (N=26), mean	*P* value (Groups 1 and 2)	*P* value (Groups 1 and 3)	*P* value (Groups 2 and 3)
**Scanning post surrogates**		20.20	15.50	25.23	<.001	<.001	<.001
	Skimming post surrogate	8.38	7.76	14.48	.74	<.001	<.001
	Examining post surrogate	11.70	7.74	10.78	<.001	.074	<.001
**Reading detailed post content**		67.17	52.14	49.39	<.001	<.001	<.001
	Skimming post content	19.64	20.40	27.36	.88	<.001	<.001
	Examining post content	47.12	31.34	21.68	<.001	<.001	<.001

For the detailed post stage, the post hoc tests showed that the participants browsing for self had longer examining duration, followed by participants browsing for others and with no issue. For the skimming duration, similar results were found as in the post surrogate stage. Participants browsing with no issue had longer skimming duration than the other two groups of participants (*P*_1,3_<.001 and *P*_2,3_<.001), whereas the other two groups of participants did not have significant difference in skimming duration (*P*_1,2_=.879).

In summary, health information seeking for self is associated with examining (especially in reading the detailed post content). This group of participants obtained the highest reading post content duration and reading-examining duration (significantly higher than that for the other two groups). It obtained medium scanning duration compared with the other two groups. It is similar to searching for others in scanning-skimming duration and similar to no issue in mind for scanning-examining duration.

Health information seeking for others is associated with short scanning duration: it obtained the shortest scanning duration and scanning-examining duration (significantly lower than that for the other two groups), and it obtained medium reading duration and reading-examining duration.

Health information browsing with no specific issue in mind is associated with skimming: it obtained the highest scanning duration, scanning-skimming duration, and reading-skimming duration, and it obtained the lowest reading duration and reading-examining duration.

### Prediction of Particular Type of User Context of Health Information Need

Stepwise multinominal logistic regression was used to develop predictive models (called classifiers) to classify a user into one of the three types of health information context, based on the browsing durations, demographic information, and urgency level. As mentioned earlier, 3 models were developed.

Two versions of model 1 were developed: model 1a made use only of the scanning post surrogates duration and model 1b made use of the scanning post surrogates and reading post content durations. Both durations can be determined by the Web application from mouse clicks on a hyperlink. Model 1a, using just the scanning duration, allows the Web application to classify the user quickly, based on the time spent on the first summary result screen. Model 1a and model 1b are shown in Tables 5 and 6 respectively, together with the classification showing their accuracy and confusion matrices.

The classification in Tables 5 and 6 indicates that model 1a has an accuracy rate of 75.7% and model 1b has an accuracy of 78.4%. However, these accuracy rates are based on the “training set.” As the sample is too small to divide into a training and a test set, 10-fold cross-validation was used to obtain a more conservative accuracy rate. In 10-fold cross validation, 10% of the participants are randomly selected from the sample to use as a validation set. The remaining 90% are used as a training set to develop a logistic regression model, which is then applied to classify the 10% validation set and calculate the accuracy rate. This is repeated 10 times with different 10% validation sets, and the mean of the 10 accuracy rates obtained are used as a conservative estimate of the accuracy rate of the final model shown in Tables 5 and 6. The 10-fold cross-validation mean accuracy rates for model 1a and model 1b (as shown in Tables 7 and 8) were 70.7% and 77.0%, respectively. The reference category is 3 (browsing with no particular issue in mind).

Model 2, as shown in Tables 9 and 10, has 3 variables: scanning duration, reading duration, and scanning-skimming duration. This obtained an accuracy of 79.7%, with a 10-fold cross validation mean accuracy of 73.9%.

Model 3, as shown in Tables 11 and 12, has 2 additional variables: age and urgency level. This model obtained an accuracy of 89.2%, with a 10-fold cross validation mean accuracy of 83.6%.

**Table 5:** Multinomial logistic regression model 1a with scanning duration only.

Type of information context		B (logistic coefficient)	Standard error	Wald	Degree of freedom	*P* value	Exp(B)	95% CI for Exp(B)
**Browsing for self**								
	Intercept	5.094	1.603	10.100	1	.001		
	Scanning_duration	−	.071	10.324	1	.001	.796	0.693-0.915
**Browsing for others**								
	Intercept	18.636	4.409	17.869	1	<.001		
	Scanning_duration	−	.248	16.506	1	<.001	.365	0.225-0.594

**Table 6:** Confusion matrix for Model 1a.

Observed	Predicted as			
	For self	For others	With no issue	Percent correct
Searching for self	21	0	4	84.0
Searching for others	6	17	0	73.9
Searching with no particular issue in mind	2	6	18	69.2
Overall percentage	39.2	31.1	29.7	75.7

**Table 7:** Multinomial logistic regression model 1b with scanning and reading duration.

Type of information context		B (logistic coefficient)	Standard error	Wald	Degree of freedom	*P* value	Exp(B)	95% CI for Exp(B)
**Browsing for self**								
	Intercept	−	3.869	4.373	1	.04		
	Scanning_duration	−	.082	1.338	1	.25	.910	0.775-1.068
	Reading_duration	.169	.050	11.578	1	.001	1.184	1.074-1.306
**Browsing for others**								
	Intercept	14.234	4.729	9.061	1	.003		
	Scanning_duration	−	.232	9.426	1	.002	.490	0.311-0.773
	Reading_duration	−	.044	.177	1	.67	.982	0.900-1.070

**Table 8:** Confusion matrix for Model 1b.

Observed	Predicted as			
	For self	For others	With no issue	Percent correct
Browsing for self	22	0	3	88.0
Browsing for others	3	19	1	82.6
Browsing with no particular issue in mind	3	6	17	65.4
Overall percentage	37.8	33.8	28.4	78.4

**Table 9:** Multinomial logistic regression model 2 with eye tracker information.

Type of information context		B (logistic coefficient)	Standard error	Wald	Degree of freedom	*P* value	Exp(B)	95% CI for Exp(B)
**Browsing**								
	Intercept	−	5.331	6.561	1	.01		
	Scanning_duration	.869	.352	6.108	1	.01	2.385	1.197-4.750
	Reading_duration	.134	.052	6.602	1	.01	1.144	1.032-1.267
	Scanning-skimming	−	.445	7.905	1	.005	.286	0.119-0.684
**Browsing**								
	Intercept	11.751	4.218	7.763	1	.005		
	Scanning_duration	−	.331	4.428	1	.03	.498	0.260-0.953
	Reading_duration	−	.046	.070	1	.79	.988	0.903-1.081
	Scanning-skimming	.205	.389	.278	1	.60	1.228	0.573-2.631

**Table 10:** Confusion matrix for Model 2.

Observed	Predicted as			
	For self	For others	With no issue	Percent correct
Searching for self	22	0	3	88.0
Searching for others	3	19	1	82.6
Searching with no particular issue in mind	3	6	17	65.4
Overall percentage	37.8	33.8	28.4	78.4

**Table 11:** Multinomial logistic regression model 3 with age and urgency information.

Type of information context		B (logistic coefficient)	Standard error	Wald	Degree of freedom	*P* value	Exp(B)	95% CI for Exp(B)
**Browsing**								
	Intercept	−	40.208	3.304	1	.07		
	Scanning duration	2.594	1.548	2.807	1	.09	13.378	0.644-278.076
	Reading duration	.339	.169	4.020	1	.045	1.403	1.008-1.954
	Scanning-skimming	−	1.490	3.042	1	.08	.074	0.004-1.379
	Urgency health	1.538	.749	4.212	1	.04	4.655	1.072-20.216
	Age	.578	.331	3.040	1	.08	1.782	0.931-3.411
**Browsing**								
	Intercept	8.984	5.022	3.201	1	.07		
	Scanning duration	−	.404	4.036	1	.045	.444	0.201-0.980
	Reading duration	−	.047	.131	1	.72	.983	0.898-1.077
	Scanning-skimming	.430	.448	.920	1	.34	1.538	.638-3.703
	Urgency health	.529	.343	2.383	1	.12	1.697	.867-3.321
	Age	.031	.069	.204	1	.65	1.031	.902-1.180

**Table 12:** Confusion matrix for Model 3.

Observed	Predicted as			
	For self	For others	With no issue	Percent correct
Searching for self	25	0	0	100.0
Searching for others	1	21	1	91.3
Searching with no particular issue in mind	0	6	20	76.9
Overall percentage	35.1	36.5	28.4	89.2

## Discussion

### Principal Findings

In this study, different types of user browsing durations in the summary results screen (listing post surrogates) and detailed post content screen of a health discussion forum were found to be associated with different types of user health information need context.

Users who are seeking information for their own health issue are more likely to closely examine the page content, especially when reading the detailed post content. Their scanning duration (in the summary results screen) is in between the other two groups. It is probably because they know their issue well and need to focus on health information related to their personal conditions during browsing [4]. Hence, they spend the longest time closely examining the detailed health information to assess whether it is related to them. However, they do not require much time to locate their health topic; hence, their skimming durations are short.

Users who are seeking information for a family member or friend’s health issue tend to have a short scanning duration in the summary results screen. They have medium reading durations in the detailed post screen between the other two groups. It is probably because they know something but not much about the health issue of their friend or relative. Hence, they are able to find their topic fairly quickly, reflected in their short skimming durations. They devote more time to examining basic information on their topic (but not the detailed health content or personal experience information), reflected in their second longest examining durations in the post content screen [4,45].

Browsing with no specific health issue in mind is associated with skimming and the longest scanning duration in the summary results screen. This group had the lowest reading duration in the post content screen. As the users do not have a particular issue in mind, they need to skim over a lot of health information to identify a topic of interest. Hence, they spent the longest time quickly skimming over to locate their interests. In contrast, their examining durations were not long as they do not devote much time to learn and digest detailed content and just read them like reading newspapers or stories [4,7].

In addition, demographic information and urgency of health information need were also found to be significant factors. Older users do more examining (with eye fixations), whereas younger users do more skimming (with quick eye movements). Users with greater health information urgency do more examining, whereas people with lower health information urgency do more skimming. Users with a higher level of formal education were found to spend more time in close reading of post content. People from different countries may exhibit different skimming and examining durations, possibly because of different levels of English reading competency.

As the Internet users have different types of health information need and information context, they naturally focus on different types of health information when browsing a health discussion forum. As the result, their examining and skimming durations differ. In turn, these durations can be used to predict the user’s particular type of health information need context. The logistic regression analysis results indicate that the accuracy of prediction can reach 90% when these durations are used together with the user’s age and urgency of health information need, as in model 3. In comparison, model 1 using only mouse click information is able to reach an accuracy of 76%.

Use of examining and skimming durations from the eye-tracking system can provide more accurate results. Model 2 that makes use of the eye tracker information, but without personal and demographic information, can reach an accuracy of 80%. This model may be feasible in the near future as mobile phones are already using retina scan, and Google glass is using eye-tracking technologies. An eye-tracking system can record examining and skimming patterns of users scanning and reading text on the screen, and software programs can be written to analyze these patterns and generate inferences about the user’s interests and context, which can be used by the health information platform to tailor health information for the user. These steps can be carried out in the background without disturbing the user’s browsing. In addition, the health information platform can request the user to create a log-in account and provide demographic and personal health information that can be used for personalization to improve the user’s browsing experience and increase the relevance of health information provided.

As the results indicate that age, education level, and nationality affect the user’s browsing duration, future research can investigate these factors in more detail as well as explore more demographic and socioeconomic variables.

### Limitations

This study was conducted at Nanyang Technological University in Singapore, and the research participants were Singapore residents. Users who are not located in the country were not included in this study.

This study focused on a health discussion forum, which has an interface and navigation structure that is similar to that of information retrieval systems and database systems, which typically have a search query screen, summary results screen, and detailed result screen. Information websites, however, have a different interface and navigation structure. The navigation structure and information organization on the screen or webpage will affect how users navigate the site and browse the displayed text.

This study recruited participants with a wide age range and included Singapore citizens as well as local residents with other nationalities (mainly China). Convenience sampling used means that the study did not cover all strata of Singapore society. In particular, residents who did not have university education were not covered. This study did not cover users with critical or severe health problems, whose browsing behaviors are likely to be different from the participants in this study. More focused studies can also be carried out to investigate differences between younger and older people and between local citizens and foreigners (including foreign students).

This study focused on health information browsing on a personal computer (PC) screen. The browsing on mobile devices was not considered. There may be differences between browsing on traditional PC and mobile devices.

## Abbreviations

ANOVA: analysis of variance
DF: degrees of freedom
PC: personal computer
SE: standard error
